# Existence of and decay to equilibrium of the filament end density along the leading edge of the lamellipodium

**DOI:** 10.1007/s00285-016-1027-z

**Published:** 2016-05-20

**Authors:** Angelika Manhart, Christian Schmeiser

**Affiliations:** 1 CIMS, New York University, 251 Mercer Street, New York, NY 10012 USA; 2 Faculty of Mathematics, University of Vienna, Oskar-Morgenstern Platz 1, 1090 Vienna, Austria

**Keywords:** Lamellipodium, Actin, Lyapunov function, 35F61, 35B35, 35B32, 35B09, 35Q92, 92C37

## Abstract

A model for the dynamics of actin filament ends along the leading edge of the lamellipodium is analyzed. It contains accounts of nucleation by branching, of deactivation by capping, and of lateral flow along the leading edge by polymerization. A nonlinearity arises from a Michaelis–Menten type modeling of the branching process. For branching rates large enough compared to capping rates, the existence and stability of nontrivial steady states is investigated. The main result is exponential convergence to nontrivial steady states, proven by investigating the decay of an appropriate Lyapunov functional.

## Introduction

The lamellipodium is a thin protrusion, developing when biological cells spread on flat surfaces. It is supported by a roughly two-dimensional meshwork of protein filaments, created by polymerization of actin (Small et al. 2002). In steadily protruding lamellipodia, the meshwork exhibits two dominant directions, approximately symmetric to the leading edge of the lamellipodium (Vinzenz et al. 2012), and can thus be approximated by two distinct families of filaments. The meshwork is a very dynamic structure, driven by the *polymerization* of the filaments abutting the leading edge, but also by the nucleation of new filaments via *branching* away from old filaments, close to their growing ends. This is responsible for the two-direction structure with the angle between the two families approximately equal to the branching angle. Finally, *capping* of filaments plays a role, whence filaments become blocked, stop to polymerize, and subsequentially lose contact to the leading edge.

**Fig. 1:**
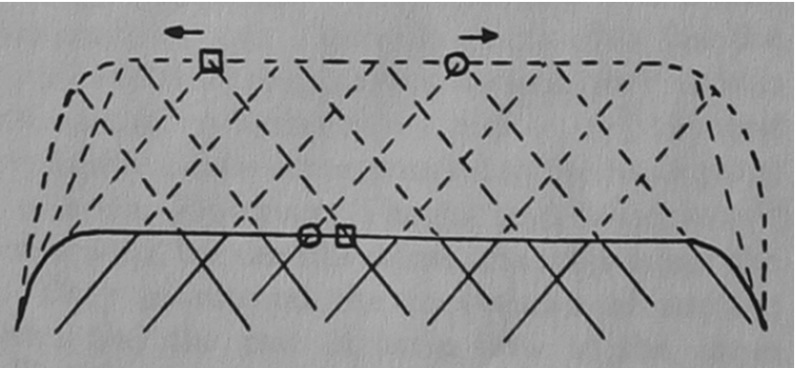
Lateral flow. *Solid* and *dashed lines* represent the present and, respectively, a future state of filaments of the leading edge (drawing courtesy of J. Vic Small)

This work deals with a model for the dynamics of filament ends along the leading edge. New filament ends are produced by branching, they disappear from the leading edge by capping, and they move along the leading edge by what is called *lateral flow,* a consequence of polymerization and of the inclination of filaments relative to the leading edge (Small 1994) (see Fig. 1). Under the idealizing assumption of a constant angle between filaments and leading edge and of a constant polymerization speed, the speed of lateral flow along the leading edge is constant. In reality, however, this cannot be expected, since the polymerization speed is subject to various influences such as chemical signaling and mechanical restrictions due to the varying geometry of the leading edge, where the latter will also lead to varying angles between filaments and leading edge.

A complete model therefore needs to describe the positions of filaments and of the leading edge. The authors have been involved in the formulation of such a modeling framework, the filament based lamellipodium model (FBLM) (Manhart et al. 2015; Oelz and Schmeiser 2010), which includes descriptions of filament bending, cross-linking, and adhesion to the substrate, as well as a number of other relevant mechanisms. The present work is concerned with a submodel describing branching and capping, and where the lateral flow speed along the leading edge will be considered as given. As a further model simplification, the lateral flow speed of both filament families will be assumed equal at each point on the leading edge. Concerning the geometry, two different situations will be considered: for cells surrounded by a lamellipodium, the leading edge is described as a one-dimensional interval with a periodicity assumption, where the two ends are identified. This situation applies mostly to stationary spreading cells and has been observed in several types of cells, such as fish keratocytes (Yam et al. 2007), mouse fibroblasts (Symons and Mitchison 1991) or T cells (Hui et al. 2012). It is important to note, that even in a situation where the cell is not moving, the lamellipodium is still very dynamic and filaments are being constantly turned over (Yam et al. 2007; Symons and Mitchison 1991). Apart from that, random fluctuations between protrusion and retraction can be observed in some cell types (Ryan et al. 2012). During the transition from a stationary to a moving cell, the rear lamellipodium typically disappears, however the periodic boundary condition would be valid up until this topological change. For a steadily moving cell, like the crawling fish keratocyte with a crescent-like shape and a lamellipodium only along the outer rim, the leading edge is represented by an interval with zero lateral inflow of filaments (see details below).

The branching process requires the Arp2/3 protein complex connecting the old and the new filament at the branch point (Svitkina and Borisy 1999). The assumptions that the availability of Arp2/3 is limiting and that the Arp2/3 dynamics is fast compared to the branching dynamics results in a Michaelis–Menten type model, similar to the one already formulated in Grimm et al. (2003). Capping is described as a simple Poisson process.

The main question of this work is: does the mathematical model describe a stable distribution of filament ends? The answer is a conditional yes with the rather obvious condition that the branching rate has to be big enough compared to the capping rate. Otherwise the filament end population dies out.

The rest of this paper is structured as follows: In Sect. 2 the derivation of the model is described. This has already been explained in the context of the full FBLM in Manhart et al. (2015), but it is also included here for the sake of completeness. In Sect. 3 an existence, uniqueness, and boundedness result is proven. It is also shown that it is enough to initially have a small amount of filament ends of only one family, to make the densities of both families positive everywhere within finite time. The short Sect. 4 is concerned with the proof of the simple result that the end distributions converge to zero, when the branching rate is too small compared to the capping rate. In Sect. 5 existence results for non-trivial stationary states are proven. There are several kinds of results. First, it is shown that a transcritical bifurcation away from the zero steady state occurs, when the ratio between the branching rate and the capping rate exceeds a critical value. This local result is extended in two special situations: In the case of the periodic leading edge, existence of a nontrivial steady state is proven also far from the bifurcation point, if the lateral flow speed is almost constant. The same result holds for the mathematically more difficult case of a leading edge with zero lateral inflow, if the lateral flow speed is constant. Finally in Sect. 6 it is shown that for every nonvanishing initial distribution the solution converges exponentially to the nontrivial steady state, if it exists.

## Derivation and nondimensionalisation of the model

A model very similar to the one considered here has been formulated in Grimm et al. (2003). We shall follow the derivation given in Manhart et al. (2015) in the framework of the FBLM.

The leading edge is assumed as a (potentially closed) rectifiable curve of length *L*, parametrized by arclength $x\in [0, L]$. We distinguish between two families of filaments, those pointing to the right with number density of ends *u*(*x*, *t*) and those pointing to the left with density *v*(*x*, *t*). By lateral flow the right-pointing filament ends are moved to the right and the left-pointing filament ends to the left, both with the prescribed position dependent speed $c(x)> 0$. It is a simplifying assumption that the speed is time independent and the same for both families. The density of activated Arp2/3 at the leading edge is denoted by *a*(*x*, *t*). Following the molecular mechanisms described e.g., in Blanchoin et al. (2000) or Pollard and Borisy (2003) we assume that prior to branch initiation, cytoplasmic Arp2/3 needs to be recruited to the leading edge, where it is activated by WASP/Scar proteins. We assume this happens with a constant rate $c_\text {rec}$, which includes both recruitment and activation of Arp2/3, and with the opposite reaction (with the rate constant written as $c_\text {rec}/a_0$) working towards the equilibrium density $a_0$ of activated, membrane associated Arp2/3. We do not go into further detail but note, that both $a_0$ and $c_\text {rec}$ depend on the availability of WASP/Scar proteins (and their activators) and the chemical properties of the activation reaction. Furthermore Arp2/3 is consumed by branching events, where new filament ends of one family create ends of the other with rate constant $\kappa _\text {br}$ (measured at the equilibrium $a_0$ of activated Arp2/3). Arp2/3 molecules at the leading edge are assumed immobile for simplicity. Finally, the rate constant for the deactivating capping reaction is denoted by $\kappa _\text {cap}$. These assumptions lead to the system

$$\begin{aligned} \partial _t u+\partial _x (c(x)u)= & {} \kappa _\text {br} \frac{a}{a_0}v-\kappa _\text {cap}u, \\ \partial _t v-\partial _x (c(x)v)= & {} \kappa _\text {br} \frac{a}{a_0}u - \kappa _\text {cap}v, \\ \partial _t a= & {} c_\text {rec} \left( 1 - \frac{a}{a_0}\right) - \kappa _\text {br} \frac{a}{a_0} (u+v). \end{aligned}$$

We introduce the scaling

$$\begin{aligned}&x\rightarrow x\,L,\quad t\rightarrow \frac{t}{\kappa _\text {cap}},\quad c(x)\rightarrow L\,\kappa _\text {cap} c(x),\\&\left( u,v\right) \rightarrow \left( u\frac{c_\text {rec}}{\kappa _\text {br}}, v\frac{c_\text {rec}}{\kappa _\text {br}}\right) , \quad a \rightarrow a_0a, \end{aligned}$$

and the dimensionless parameters

$$\begin{aligned} \alpha :=\frac{\kappa _\text {br}}{\kappa _\text {cap}},\quad \varepsilon = \frac{\kappa _\text {cap} a_0}{c_\text {rec}}, \end{aligned}$$

where $\alpha$, the ratio between the branching and the capping rates, is assumed of moderate size, whereas $\varepsilon$, the ratio between the characteristic time for Arp2/3 and that of the capping and branching processes will be assumed as small. This assumption can also be interpreted as smallness of the ratio $\varepsilon \alpha$ between the reference values $a_0$ for the Arp2/3 density and $c_\text {rec}/\kappa _\text {br}$ for the filament end densities, with the consequence that the availability of activated Arp2/3 is limiting for the branching process.

Whereas the first assumption is justified and actually necessary, as our analysis will show, the smallness of $\varepsilon$ has, to the knowledge of the authors, not been verified experimentally. The nondimensionalized system has the form

$$\begin{aligned} \partial _t u+\partial _x (c(x)u)= & {} \alpha av - u,\\ \partial _t v-\partial _x (c(x)v)= & {} \alpha au - v,\\ \varepsilon \partial _t a= & {} 1 - a(1+u+v). \end{aligned}$$

The last step in the model derivation is to pass to the quasistationary limit $\varepsilon \rightarrow 0$ in the equation for *a*, which is analogous to the derivation of Michaelis–Menten kinetics. Elimination of *a* from the resulting system gives

1 $$\begin{aligned}&\partial _t u + \partial _x \left( c(x) u\right) =\frac{\alpha \, v}{1+u+v}-u, \nonumber \\&\partial _t v - \partial _x \left( c(x) v\right) =\frac{\alpha \, u}{1+u+v}-v, \end{aligned}$$

for $x\in [0,1]$. Two types of boundary conditions are biologically relevant. In the case of a ring-shaped lamellipodium around the whole cell we assume periodic boundary conditions. On the other hand, if we consider only a lamellipodium at the front, it is reasonable to assume that no left-moving filaments enter from the right and vice versa. These considerations allow to complement (1) with one of the following sets of boundary conditions:

2 $$\begin{aligned} \text {(DBC)} \quad u(0,t)= & {} 0, \quad v(1,t)=0, \quad \text {for } \; t > 0, \end{aligned}$$

3 $$\begin{aligned} \text {(PBC)}\quad u(0,t)= & {} u(1,t), \quad v(0,t)=v(1,t), \quad \text {for } \; t > 0. \end{aligned}$$

Throughout this paper, we will use the abbreviations (*DBC*) and (*PBC*) for Dirichlet Boundary Conditions and Periodic Boundary Conditions, respectively. For (*PBC*) we implicitly assume that also the lateral flow speed *c*(*x*) is periodic. To complete the definition of the problem, we pose initial conditions

4 $$\begin{aligned} u(x,0)=u_0(x), \quad v(x,0)=v_0(x), \quad \text {for } \; x\in [0,1], \end{aligned}$$

with given $u_0(x), v_0(x)$, which are assumed to be non-negative and to satisfy the boundary conditions.

## Existence, uniqueness, and positivity of solutions

We start with a reformulation of the problem, which will be useful in most of our proofs. It is based on the assumption that the lateral flow speed is not only positive, but bounded away from zero: There exist positive constants $\underline{c},\overline{c}$, such that

5 $$\begin{aligned}&0 < \underline{c}\le c\le \overline{c} \quad \text{ in } [0,1]. \end{aligned}$$

An average inverse speed can then be defined by

$$\begin{aligned} \frac{1}{C} = \int _0^1 \frac{\,\mathrm {d}x}{c(x)}. \end{aligned}$$

Now we introduce the transformation $(x,u,v) \leftrightarrow (X,U,V)$ by

6 $$\begin{aligned} X = C\int _0^x \frac{\,\mathrm {d}\xi }{c(\xi )} \in [0,1],\quad cu = CU,\quad cv = CV. \end{aligned}$$

The transformed version of (1) reads

7 $$\begin{aligned}&\partial _t U + C\partial _X U = R_{U,V}-U,\nonumber \\&\partial _t V - C \partial _X V = R_{V,U}-V, \end{aligned}$$

with

$$\begin{aligned} R_{U,V}(X,t) = \frac{\alpha \, V(X,t)}{1+\beta (X)(U(X,t)+V(X,t))}, \end{aligned}$$

and $\beta = C/c$, bounded from above and below by

8 $$\begin{aligned}&0< \underline{\beta }:= C/\overline{c} \le \beta \le C/\underline{c} =: \overline{\beta }\quad \text{ in } [0,1]. \end{aligned}$$

Note that (*U*, *V*) satisfies the same boundary conditions (2), (3) as (*u*, *v*). The transformed initial conditions read

9 $$\begin{aligned} U(t=0)=U_0:=\frac{c u_0}{C}, \quad V(t=0)=V_0:=\frac{c v_0}{C},\quad \text {in } [0,1]. \end{aligned}$$

The reformulation has two effects: First, the solution of the equations by the method of characteristics is simplified, since the transformed lateral flow speed is constant, and, second, linearization around the zero solution gives a problem with constant coefficients. Applying the method of characteristics leads to a mild formulation.

### Definition 1

(PBC) Let $(U,V)\in {\mathcal {C}}(\mathbb {T}^1\times [0,\infty ))^2$ (with the torus $\mathbb {T}^1$ represented by the interval [0, 1]) satisfy

10 $$\begin{aligned} U(X,t)= & {} U_0(X-Ct)e^{-t} + \int _0^t e^{s-t} R_{U,V}(X+C(s-t),s)\,\mathrm {d}s,\nonumber \\ V(X,t)= & {} V_0(X+Ct)e^{-t} + \int _0^t e^{s-t} R_{V,U}(X-C(s-t),s)\,\mathrm {d}s, \end{aligned}$$

for $$(X,t)\in \mathbb {T}^1\times [0,\infty )$$. Then

11 $$\begin{aligned} (u(x,t),v(x,t)) = \frac{C}{c(x)} (U(X(x),t),V(X(x),t)) \end{aligned}$$

is called a global mild solution of the problem (1), (3), (4). (DBC) Let $(U,V)\in {\mathcal {C}}([0,1]\times [0,\infty ))^2$ satisfy

12 $$\begin{aligned} \begin{aligned} U(X,t)&= U_0(X-Ct)H(X-Ct)e^{-t}\\&\quad \ + \int _{(t-X/C)_+}^t e^{s-t} R_{U,V}(X+C(s-t),s)\,\mathrm {d}s,\\ V(X,t)&= V_0(X+Ct)H(1-X-Ct)e^{-t}\\&\quad \ + \int _{(t-(1-X)/C)_+}^t e^{s-t} R_{V,U}(X-C(s-t),s)\,\mathrm {d}s, \end{aligned} \end{aligned}$$

for $(X,t)\in [0,1]\times [0,\infty )$, where *H* denotes the Heavyside function. Then (*u*, *v*) defined by (11) is called a global mild solution of the problem (1), (2), (4).

### Proposition 1

Let $u_0, v_0, c\in {\mathcal {C}}([0,1])$ satisfy (5), $u_0,v_0\ge 0$, and the boundary conditions (DBC) or (PBC). Then the problem (1), (2), (4), or, respectively, (1), (3), (4), has a unique, global mild solution (*u*, *v*), satisfying

13 $$\begin{aligned} 0\le u(x,t) \le \frac{\overline{c}}{\underline{c}} \max \left\{ \max _{[0,1]} u_0, \alpha \right\} ,\quad 0\le v(x,t) \le \frac{\overline{c}}{\underline{c}} \max \left\{ \max _{[0,1]} v_0, \alpha \right\} . \end{aligned}$$

### Remark 1

It is straightforward to show by differentiation of the equations that for smooth initial data $u_0,v_0\in {\mathcal {C}}^\infty (\mathbb {T}^1)$ for (PBC), and $u_0\in {\mathcal {C}}^\infty _0((0,1])$, $v_0\in {\mathcal {C}}^\infty _0([0,1))$ for (DBC), and for smooth lateral flow speed $c\in {\mathcal {C}}^\infty ([0,1])$, the solution satisfies $u,v\in {\mathcal {C}}^\infty ([0,1]\times [0,\infty ))$. Some results of the following sections are based on computations with the strong forms (1) or (7) of the differential equations. These can be justified by uniform smooth approximations of $u_0,v_0,c$, and subsequent removal of the smoothing.

### Proof

The (obvious) non-negativity and Lipschitz continuity of $R_{U,V}$ in terms of $(U,V)\in [0,\infty )^2$, as well as the bound $R_{U,V}\le \alpha /\underline{\beta }$ will be sufficient for carrying out the proof.

With the Lipschitz continuity, it is straightforward to show that the right hand sides of (10) and (12) preserve non-negativity and are contractions on ${\mathcal {C}}(\mathbb {T}\times [0,T])$ and, respectively, ${\mathcal {C}}([0,1]\times [0,T])$, for *T* small enough, which proves local existence. The estimate

$$\begin{aligned} 0\le u(x,t)&\le \frac{C}{\underline{c}}\left( e^{-t} \sup _{[0,1]} U_0 + \frac{\alpha }{\underline{\beta }} (1-e^{-t})\right) \le \frac{C}{\underline{c}}\max \left\{ \sup _{[0,1]} U_0, \frac{\alpha }{\underline{\beta }} \right\} \\&\le \frac{\overline{c}}{\underline{c}} \max \left\{ \max _{[0,1]} u_0, \alpha \right\} \end{aligned}$$

and the analogous version for *v* allow to continue the local solution indefinitely and also prove (13). $\square$

One expects that if the initial conditions $(u_0(x), v_0(x))$ are positive [except at the boundaries for (*DBC*)], the same holds for the solution (*u*(*x*, *t*), *v*(*x*, *t*)) for all $t\ge 0$. In fact a much stronger result is true: it is enough to have positivity of the initial data on some interval for only one family; after finite time both families will be positive everywhere [except at the boundaries for (*DBC*)]. The reason for this is that, although the initial mass is reduced by capping while it is transported across the domain, it remains positive. This mass, however, will trigger the creation of new filaments of the other family through the branching term. This new mass will be transported in the opposite direction and will itself cause the creation of mass of the first family. As long as the lateral flow speed *c*(*x*) is bounded from below, this process happens in finite time.

### Proposition 2

Let the assumptions of Proposition 1 hold and let the initial data satisfy

14 $$\begin{aligned} \int _0^1(u_0+v_0)\,\mathrm {d}x > 0. \end{aligned}$$

Then, for every $T>2\int _0^1 \,\mathrm {d}x/c(x)$,

$$\begin{aligned}&\text{ for } \text{(PBC): }\quad u,v>0\quad \text {in } [0,1]\times [T,\infty ), \\&\text{ for } \text{(DBC): }\quad u>0\quad \text {in } (0,1]\times [T,\infty ),\quad v>0\quad \text {in } [0,1)\times [T,\infty ). \end{aligned}$$

**Fig. 2:**
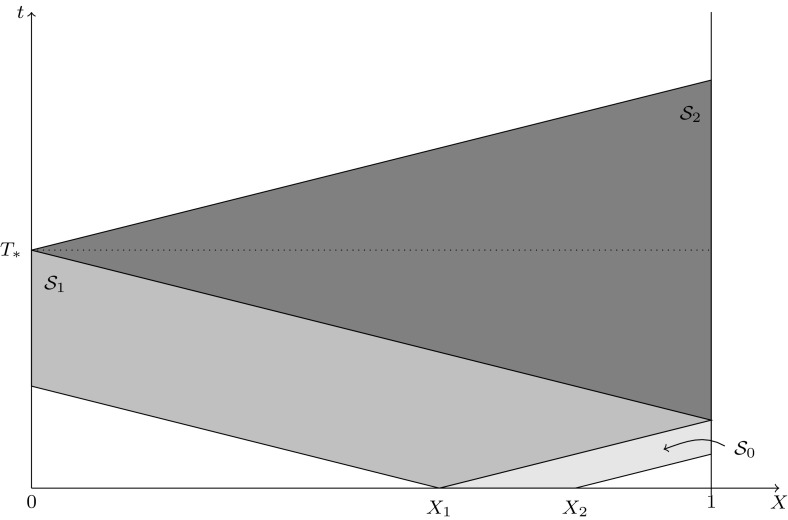
Illustration of the proof of Proposition 2

### Proof

The equivalent result for (*U*, *V*) will be proved. Assumption (14) and the continuity of the initial data imply the existence of an interval $[X_1,X_2]\subset [0,1]$ of positive length and of $m>0$, such that one of the initial densities, w.l.o.g. $U_0$, satisfies $U_0 \ge m$ in $[X_1,X_2]$.

The mild formulations given in Definition 1 show that, whenever a *U*-characteristic (with velocity *C*) passes through a region where *V* is positive, *U* is positive along this characteristic in and after (timewise) this region, and vice versa ($U\leftrightarrow V$, $C\leftrightarrow -C$). Also *U* is positive everywhere along a *U*-characteristic after a point on the characteristic, where it is positive (again the same for *V*).

These observations reduce the proof to a geometric problem (see Fig. 2), where we alternate between using the equations for *U* and for *V*. The first step is the observation that by the above property of the initial data, *U* is positive in the strip $\mathcal {S}_0$, defined by $X_1< X-Ct < X_2$ (light gray shading in Fig. 2). This implies, as the second step, that *V* is positive there and along all *V*-characteristics starting in this strip, i.e. also in the region $\mathcal {S}_1$ bounded by $X=0$, $X-Ct=X_1$, $X+Ct=X_1$, $X+Ct=2-X_1$ (gray shading in Fig. 2). The third step is to draw again *U* characteristics, now starting in $\mathcal {S}_1$, which adds a triangle $\mathcal {S}_2$ (dark gray shading in Fig. 2) above $\mathcal {S}_1$, where $U>0$. Straightforward continuation shows that for $t\ge T_*=(1-X_1)/C+1/C$, *U* and *V* are positive for $0<X<1$. Positivity on the boundary, except for $U(0,t)=V(1,t)=0$ in the (DBC) case, is achieved at any time after $T_*$, implying that the result of the proposition holds with any $T>2/C\ge T_*$. $\square$

## When capping exceeds branching

The dimensionless parameter $\alpha$ is the ratio between the branching and the capping rate. If it is too small, it can be expected that *u* and *v* tend to zero as $t\rightarrow \infty$. Note that, on the other hand, Proposition 2 holds for all $\alpha >0$. This means that even if we start with initial conditions, which are zero everywhere and are only positive for one family in a small interval, the solutions first become positive everywhere before they decay to zero.

### Proposition 3

Let the assumptions of Proposition 1 and $0\le \alpha <1$ hold. Then the solution (*u*, *v*) of (1), (2), (4) or (1), (3), (4) satisfies

$$\begin{aligned} \Vert u(\cdot ,t)\Vert _{L^2((0,1))} + \Vert v(\cdot ,t)\Vert _{L^2((0,1))} \le e^{(\alpha -1)t} \left( \Vert u_0\Vert _{L^2((0,1))} + \Vert v_0\Vert _{L^2((0,1))}\right) . \end{aligned}$$

### Proof

We observe that

$$\begin{aligned} \frac{\,\mathrm {d}}{\,\mathrm {d}t}\int _0^1 \frac{1}{2}\left( u^2+v^2\right) \,\mathrm {d}x=&\int _0^1 \left( \frac{2\alpha uv}{1+u+v} - u^2 - v^2\right) \,\mathrm {d}x \\&+{\left\{ \begin{array}{ll} 0 &{}\quad \text {(PBC)}, \\ -c(1)u(1,t)-c(0)v(0,t) &{}\quad \text {(DBC)}. \end{array}\right. } \end{aligned}$$

The non-positivity of the boundary term and the estimate

$$\begin{aligned} \frac{2\alpha uv}{1+u+v} - u^2 - v^2= & {} \frac{(\alpha -1)(u^2+v^2) - \alpha (u-v)^2 - (u+v)(u^2+v^2)}{1+u+v} \\\le & {} (\alpha -1) \left( u^2+v^2\right) \end{aligned}$$

finish the proof. $\square$

The results of the following sections show that the bound on $\alpha$ is sharp for (PBC), but not for (DBC), where decay to zero can be expected also for $1\le \alpha < \alpha _0$ with the bifurcation value $\alpha _0$.

## Existence of nontrivial steady states

### Bifurcation from the zero solution

A bifurcation value $\alpha _0$ for the ratio $\alpha$ between the branching rate and the capping rate will be computed, where a nontrivial steady state branches off the zero solution. By repeating the computation in the proof of Proposition 3 for the steady state problem, proving that for $\alpha <1$ it has only the zero solution. Therefore we expect $\alpha _0\ge 1$, with the zero solution being the stable equilibrium for $\alpha \le \alpha _0$ and with a second steady state existing for $\alpha >\alpha _0$, whence the zero solution is unstable and the new steady state is the stable equilibrium.

We shall work again with the variables (*X*, *U*, *V*), where the linearization around the zero solution has constant coefficients. We rewrite (7) as

15 $$\begin{aligned} \partial _t \begin{pmatrix} U \\ V \end{pmatrix} = {\mathcal {L}}_\alpha (U,V) - \frac{\alpha \beta (U+V)}{1+\beta (U+V)} \begin{pmatrix} V \\ U\end{pmatrix}, \end{aligned}$$

with the linearized operator

$$\begin{aligned} {\mathcal {L}}_\alpha (U,V) = \begin{pmatrix} {\mathcal {L}}_{\alpha ,1} (U,V) \\ {\mathcal {L}}_{\alpha ,2} (U,V) \end{pmatrix} = \begin{pmatrix} \alpha V - U - C\partial _X U \\ \alpha U - V + C\partial _X V \end{pmatrix}. \end{aligned}$$

The bifurcation point will be the smallest value $\alpha _0\ge 1$, where ${\mathcal {L}}_{\alpha _0}$, subject to the boundary conditions (2) or (3), has a nontrivial null space.

By inspection it is obvious that for (PBC) $\alpha _0=1$ holds with $({\tilde{U}},{\tilde{V}}) := (1,1) \in \mathcal {N}({\mathcal {L}}_1)$. Straightforward computations show that the null space is one-dimensional.

For (DBC) another straightforward, although a little longer computation gives that bifurcation values are of the form $\alpha = 1/|\cos b|$, where *b* solves

16 $$\begin{aligned} Cb + \tan b = 0. \end{aligned}$$

We denote by $b_0\in (\pi /2,\pi )$ the smallest positive solution, set $\alpha _0 = 1/|\cos b_0|$, and note that

17 $$\begin{aligned} \begin{pmatrix} {\tilde{U}} \\ {\tilde{V}} \end{pmatrix} := \begin{pmatrix} \sin (b_0 X) \\ \sin (b_0(1-X)) \end{pmatrix} \in \mathcal {N}({\mathcal {L}}_{\alpha _0}). \end{aligned}$$

Note that ${\tilde{U}}>0$ in (0, 1] and ${\tilde{V}} > 0$ in [0, 1). Again the null space is one-dimensional.

For both types of boundary conditions, infinite increasing sequences of bifurcation values exist. However, for all bifurcation values larger than $\alpha _0$, the null spaces, and therefore the bifurcating solutions, consist of functions with changing signs, which are irrelevant for our application and have no chance to be the long-time limit of non-negative solutions.

The normal form reduction close to the bifurcation point is derived by choosing values of $\alpha$ close to $\alpha _0$ and by making the ansatz

18 $$\begin{aligned} \begin{pmatrix} U(X,t) \\ V(X,t) \end{pmatrix} = (\alpha -\alpha _0)B(t) \begin{pmatrix} {\tilde{U}}(X) \\ {\tilde{V}}(X) \end{pmatrix} + \mathcal {O}((\alpha -\alpha _0)^2), \end{aligned}$$

expressing the fact that we expand around the zero solution for $\alpha$ close to $\alpha _0$, and the expectation that the leading term of the perturbation is in the direction of the steady states of the linearized problem. For the linearized operator and its formal adjoint with respect to the scalar product in $L^2((0,1))^2$, the symmetry property

$$\begin{aligned} {\mathcal {L}}_\alpha ^*(U,V) = \begin{pmatrix} {\mathcal {L}}_{\alpha ,2} (V,U) \\ {\mathcal {L}}_{\alpha ,1} (V,U) \end{pmatrix} \end{aligned}$$

holds. Thus, the scalar product of (15), written in the form

$$\begin{aligned} \partial _t \begin{pmatrix} U \\ V \end{pmatrix} = {\mathcal {L}}_{\alpha _0} (U,V) + \left( \alpha -\alpha _0 - \frac{\alpha \beta (U+V)}{1+\beta (U+V)} \right) \begin{pmatrix} V \\ U \end{pmatrix}, \end{aligned}$$

with $$({\tilde{V}},{\tilde{U}})$$ gives

$$\begin{aligned} \frac{\,\mathrm {d}}{\,\mathrm {d}t} \int _0^1 (U{\tilde{V}} + V{\tilde{U}})\,\mathrm {d}X = \int _0^1 \left( \alpha -\alpha _0 - \frac{\alpha \beta (U+V)}{1+\beta (U+V)} \right) (V{\tilde{V}} + U{\tilde{U}})\,\mathrm {d}X. \end{aligned}$$

Substitution of the ansatz (18) leads to

19 $$\begin{aligned} \frac{\,\mathrm {d}B}{\,\mathrm {d}t} = (\alpha - \alpha _0) B(\kappa _1 - a\kappa _2) + \mathcal {O}((\alpha -\alpha _0)^2), \end{aligned}$$

with

$$\begin{aligned} \kappa _1 = \frac{\int _0^1({\tilde{U}}^2 + {\tilde{V}}^2)\,\mathrm {d}X}{2\int _0^1 {\tilde{U}} {\tilde{V}} \,\mathrm {d}X}> 0,\quad \kappa _2 = \frac{\alpha _0\int _0^1\beta ({\tilde{U}} + {\tilde{V}})({\tilde{U}}^2 + {\tilde{V}}^2)\,\mathrm {d}X}{2\int _0^1 {\tilde{U}} {\tilde{V}} \,\mathrm {d}X} > 0. \end{aligned}$$

This is the normal form of the transcritical bifurcation. It indicates that for $\alpha <\alpha _0$ the trivial steady state is stable, whereas for $\alpha >\alpha _0$, stability is transferred to the bifurcating steady state

20 $$\begin{aligned} \begin{pmatrix} {\bar{U}}\\ {\bar{V}}\end{pmatrix} = (\alpha -\alpha _0)\frac{\kappa _1}{\kappa _2}\begin{pmatrix} {\tilde{U}}\\ {\tilde{V}}\end{pmatrix} + \mathcal {O}((\alpha -\alpha _0)^2). \end{aligned}$$

This result is of course only formal. For the steady state bifurcation, however, our computations, in particular $\kappa _1,\kappa _2\ne 0$, verify the conditions of the Crandall-Rabinowitz theory (Crandall and Rabinowitz 1971) for bifurcations from a simple eigenvalue. Without going into further detail, we shall state the result below. We do not attempt to rigorously justify the dynamic normal form reduction (19), since the stability of the bifurcating state will be proved directly in the following section.

#### Proposition 4

Let (5) hold and let $\alpha _0$ be defined as above. Then there exists $\alpha _1>\alpha _0$, such that the system (1) with (2) or (3) has a smooth (with respect to $\alpha$) branch of nontrivial solutions $({\bar{u}}, {\bar{v}}): [\alpha _0,\alpha _1)\rightarrow {\mathcal {C}}([0,1])^2$ with $({\bar{u}},{\bar{v}})\bigm |_{\alpha =\alpha _0} = (0,0)$ and with

$$\begin{aligned} \frac{\,\mathrm {d}}{\,\mathrm {d}\alpha }\begin{pmatrix}{\bar{u}}\\ {\bar{v}}\end{pmatrix}(x)\Bigm |_{\alpha =\alpha _0} = \frac{\kappa _1 C}{\kappa _2 c} \begin{pmatrix} {\tilde{U}},{\tilde{V}} \end{pmatrix} \left( C\int _0^x \frac{\,\mathrm {d}y}{c(y)}\right) . \end{aligned}$$

In a neighborhood of the point $(u,v,\alpha )=(0,0,\alpha _0)$ in ${\mathcal {C}}([0,1])^2\times \mathbb {R}$ no other stationary solutions besides the trivial solution and the solutions on the nontrivial branch exist.

#### Lemma 1

Let the assumptions of Proposition 4 hold. Then for $\alpha -\alpha _0>0$ small enough,

$$\begin{aligned}&\text{ for } \text{(PBC): }\quad {\bar{u}},{\bar{v}}> 0 \quad \text{ in } [0,1],\\&\text{ for } \text{(DBC): }\quad {\bar{u}}> 0 \quad \text{ in } (0,1],\quad {\bar{v}} > 0 \quad \text{ in } [0,1). \end{aligned}$$

#### Proof

Proposition 4 justifies (20), with $\mathcal {O}((\alpha -\alpha _0)^2)$ to be understood uniformly in $X\in [0,1]$. This immediately implies the result, except for the case of (DBC), where the behavior of ${\bar{U}}$ close to $X=0$ and of ${\bar{V}}$ close to $X=1$ has to be examined. However,

$$\begin{aligned} {\bar{U}}(0) = 0,\quad \frac{\,\mathrm {d}{\bar{U}}}{\,\mathrm {d}X}(0) = \frac{\alpha }{C}{\bar{V}}(0) >0,\quad {\bar{V}}(1) = 0,\quad \frac{\,\mathrm {d}{\bar{V}}}{\,\mathrm {d}X}(1) = -\frac{\alpha }{C}{\bar{U}}(1) < 0, \end{aligned}$$

implies positivity of ${\bar{U}}$ near $X=0$ and of ${\bar{V}}$ near $X=1$, completing the proof. $\square$

### Periodic boundary conditions: almost constant lateral flow speed

In the case of periodic boundary conditions and of a constant lateral flow speed, the bifurcating branch of nontrivial solutions is given by

21 $$\begin{aligned} \begin{pmatrix}{\bar{u}}(x)\\ {\bar{v}}(x)\end{pmatrix} = \frac{\alpha -1}{2}\begin{pmatrix}1\\ 1\end{pmatrix} ,\quad \alpha >1. \end{aligned}$$

So it is explicit, homogeneous, and global. We make use of these properties to construct a global branch for almost constant lateral flow speeds, satisfying

$$\begin{aligned} c(x) = c_0 + \varepsilon c_1(x), \end{aligned}$$

with a constant $c_0>0$, with a smooth function $c_1(x)$, and with a small parameter $\varepsilon$. Substitution of the transformation

$$\begin{aligned} \begin{pmatrix}{\bar{u}}(x)\\ {\bar{v}}(x)\end{pmatrix} = \frac{\alpha -1}{2}\begin{pmatrix}1\\ 1\end{pmatrix} + \varepsilon (\alpha -1) \begin{pmatrix} u_1(x)\\ v_1(x)\end{pmatrix} \end{aligned}$$

in (1) gives

22 $$\begin{aligned} \frac{\,\mathrm {d}}{\,\mathrm {d}x} \begin{pmatrix}u_1\\ v_1\end{pmatrix}-M(\alpha )\begin{pmatrix}u_1\\ v_1\end{pmatrix} = h(x) + \varepsilon r(u_1,v_1,x;\alpha ,\varepsilon ), \end{aligned}$$

with

23 $$\begin{aligned} M(\alpha ) =\frac{1}{2 c_0\alpha }\begin{pmatrix} 1-3\alpha &{}&{} \alpha +1 \\ -\alpha -1 &{}&{} 3\alpha -1 \end{pmatrix}, \quad h(x)=-\frac{c_1'(x)}{2c_0}\begin{pmatrix} 1 \\ 1 \end{pmatrix}, \end{aligned}$$

and where *r* is smooth in all its variables and uniformly bounded in terms of $\alpha \rightarrow \infty$ and $\varepsilon \rightarrow 0$ with commuting limits. It also satisfies

24 $$\begin{aligned} r(\alpha =1) = \tilde{r}(u_1,v_1,x;\varepsilon )\begin{pmatrix}1\\ 1\end{pmatrix}. \end{aligned}$$

These observations are the result of a lengthy but straightforward computation. The next step is to rewrite (22) as a fixed point problem by inverting the linear operator on the left hand side on the space of periodic functions. An explicit computation gives

25 $$\begin{aligned} \begin{pmatrix} u_1(x;\alpha ,\varepsilon ) \\ v_1(x;\alpha ,\varepsilon ) \end{pmatrix}&= e^{Mx} \left( \left( e^{-M}-\mathbb {I}\right) ^{-1}\int _0^1 e^{-My}(h+\varepsilon r)(y) \,\mathrm {d}y\right. \\&\qquad \left. +\int _0^x e^{-My}(h+\varepsilon r)(y)\,\mathrm {d}y\right) \nonumber , \end{aligned}$$

requiring the existence of the inverse of $e^{-M}-\mathbb {I}$, equivalent to the invertibility of *M*, which is true for $\alpha >1$. Since $M(\alpha )$ also converges to an invertible matrix as $\alpha \rightarrow \infty$, the linear operator applied to $h+\varepsilon r$ on the right hand side is uniformly bounded in $\alpha \in [\alpha ^{*},\infty )$ with $\alpha ^*>1$. Obviously, for $\varepsilon$ small enough, solutions can be constructed by contraction on the space ${\mathcal {C}}_B(\mathbb {T}^1\times [\alpha ^*,\infty ))^2$ of bounded continuous functions of the periodic variable *x* and of the parameter $\alpha$. We skip the details of the proof.

#### Proposition 5

Let $\alpha ^*>1$ and let

$$\begin{aligned} c(x)=c_0+\varepsilon c_1(x),\quad \text{ with } \quad c_0>0,\quad c_1 \in {\mathcal {C}}^1(\mathbb {T}^1). \end{aligned}$$

Then there exist $\varepsilon _0,C>0$ such that for every $\varepsilon \le \varepsilon _0$ and for every $\alpha \ge \alpha ^*$, the problem (1), (3) with (PBC) has a smooth stationary solution $({\bar{u}},{\bar{v}})$, satisfying

$$\begin{aligned}&\Vert {\bar{u}}-(\alpha -1)(1/2 + \varepsilon u_1(\cdot \,;\alpha ,0))\Vert _{L^\infty ([0,1])} + \\&\Vert {\bar{v}}-(\alpha -1)(1/2 + \varepsilon v_1(\cdot \,;\alpha ,0))\Vert _{L^\infty ([0,1])}\le \varepsilon ^2 \alpha C, \end{aligned}$$

where $(u_1(x;\alpha ,0), v_1(x;\alpha ,0))\in {\mathcal {C}}_B(\mathbb {T}^1\times [\alpha ^*,\infty ))^2$ is given by (25) with $\varepsilon =0$.

**Fig. 3:**
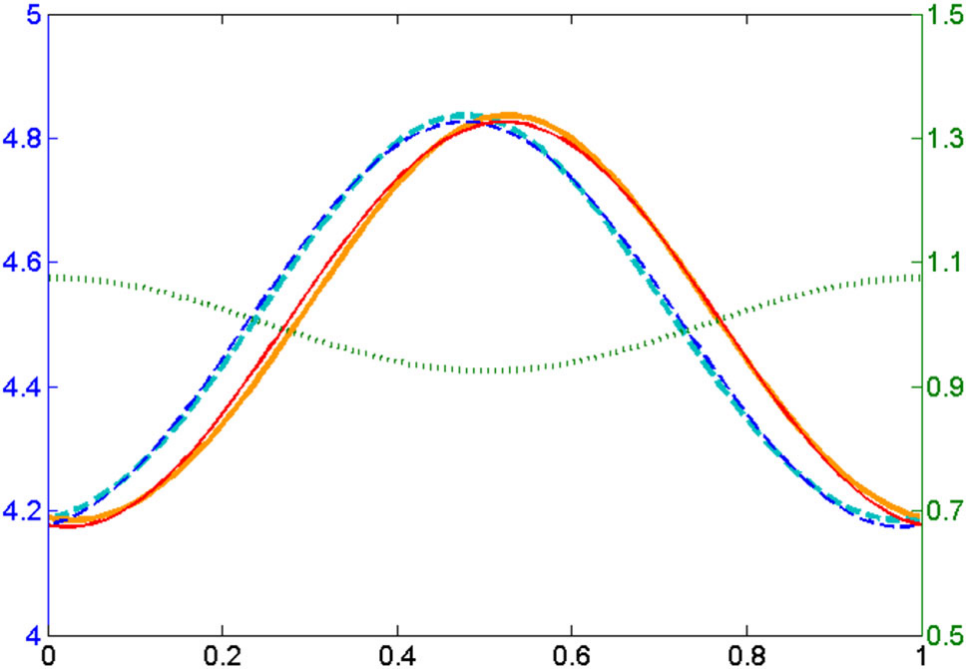
Approximate and numerical solution for $\alpha =10$ and $\varepsilon =0.1$ with *c*(*x*) given by (26). The end density of the *right* moving filaments *u* is depicted in *dark* and *light blue* (*dashed*) and that of the *left* moving filaments *v* (*solid*) in *red* and *orange*. *Thin lines* (*red* and *dark blue*) are the asymptotic approximations, *thick lines* (*orange* and *light blue*) are the numerical solution of the time dependent problem after $t=20$ and the left *y*-axis applies. In *green* (*dotted*) the lateral flow *c*(*x*) is depicted, the values are in relation to the right *y*-axis

#### Remark 2

Note the uniformity of the result in terms of $\alpha \in [\alpha ^*,\infty )$. In particular the upper bound $\varepsilon _0$ for $\varepsilon$ and the constant *C* in the error estimate are independent from $\alpha \rightarrow \infty$. There is some subtlety to the situation as $\alpha \rightarrow 1$, since the matrix $(e^{-M(\alpha )} - \mathbb {I})^{-1}$ blows up in this limit. However the limiting right hand sides being proportional to the vector (1, 1) [see (23), (24)] satisfy the solvability conditions for $\alpha =1$, so that a bounded passage to the limit can be expected. We do not carry out this limit in detail, since it is roughly equivalent to the bifurcation analysis above.

We conclude with an example: setting

26 $$\begin{aligned} c(x)=1+\frac{3\varepsilon }{4} \cos (2\pi x) \end{aligned}$$

yields

27 $$\begin{aligned}&u_1(x;\alpha ,0)=-\frac{3\pi \left( 2\alpha \pi \cos (2\pi x)\right) -(\alpha -1)\sin (2\pi x)}{8 \left( \alpha -1+2\pi ^2\alpha \right) },\nonumber \\&v_1(x;\alpha ,0)=-\frac{3\pi \left( 2\alpha \pi \cos (2\pi x)\right) +(\alpha -1)\sin (2\pi x)}{8 \left( \alpha -1+2\pi ^2\alpha \right) }. \end{aligned}$$

Figure 3 shows the approximate steady state solution

$$\begin{aligned} (\alpha -1)(1/2 + \varepsilon u_1(x;\alpha ,0), 1/2 + \varepsilon v_1(x;\alpha ,0)) \end{aligned}$$

together with a numerical solution computed as a steady state of the time dependent problem. The lateral flow in the example corresponds qualitatively to a simulation with the full filament based lamellipodium model (Manhart et al. 2015), where the cell is moving and the front is located at $x=0$ (identified with $x=1$) and the back at $x=0.5$. The lateral flow speed *c*(*x*) has its minimum at the back and its maximum at the front. The steady state distributions of both filament families have their maximum at the back, due to the accumulation of filaments by the lateral flow. The maximum of the right-moving family is shifted slightly to the left as compared to the cell rear and vice versa for the left-moving filaments. The reason for this seemingly counter-intuitive result is that filaments of the right-moving family are produced by left-moving filaments, which shifts the maximum of the right-moving family to the left.

### Dirichlet boundary conditions: constant lateral flow speed

In terms of the new unknowns $p={\bar{u}}+{\bar{v}}$, $q={\bar{u}}-{\bar{v}}$, the stationary version of (1) with constant lateral flow speed *c* can be written as

28 $$\begin{aligned} c p^{\prime } = -q\left( 1+ \frac{\alpha }{1+p}\right) ,\quad cq^\prime = p\left( \frac{\alpha }{1+p} - 1\right) . \end{aligned}$$

The boundary conditions (DBC) translate to

$$\begin{aligned} p(0)+q(0) = 0,\quad p(1)-q(1) = 0. \end{aligned}$$

The bifurcating solutions constructed above have the symmetry

$$\begin{aligned} {\bar{u}}(x) = {\bar{v}}(1-x) \quad \longleftrightarrow \quad p(x) = p(1-x),\quad q(x) = -q(1-x), \end{aligned}$$

and we shall look for solutions with this property. This allows to reduce the problem to the interval [0, 1 / 2] with the boundary conditions

$$\begin{aligned} p(0)+q(0) = 0,\quad q(1/2) = 0. \end{aligned}$$

**Fig. 4:**
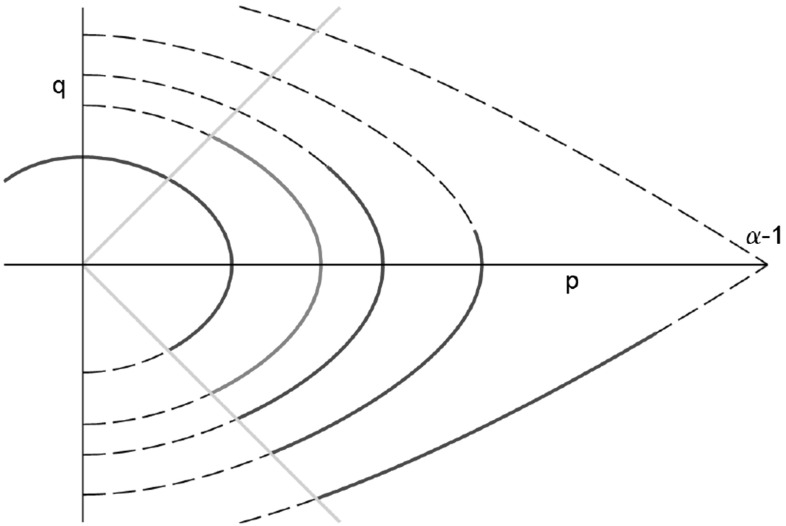
This figure shows the trajectories in (*p*, *q*)-phase space of the stationary equation with constant *c* and (*DBC*). The *orange*, *diagonal lines* represent the boundary conditions $q=-p$ at $x=0$ and $q=p$ at $x=1$. *Dashed curves* represent (29) for different values of $E_0$ and the *blue lines* the corresponding solutions curves fulfilling $p(0)+q(0)=0$. The *red line* is desired the solution satisfying both boundary conditions

Viewing (28) as a dynamical system and assuming $\alpha >1$, it has the critical points $(p,q)=(0,0)$, which is a center, and the saddle $(p,q)=(\alpha -1,0)$. We look for solutions following trajectories in the region bounded by $p+q=0$, by $q=0$, and by the stable manifold of the saddle. The system has the first integral

29 $$\begin{aligned}&E_0 = q^2 + E(p,\alpha ),\quad \text{ with }\nonumber \\&E(p,\alpha ) = - p^2 + 4\alpha p - 4\alpha (\alpha +1)\ln \left( 1 + \frac{p}{\alpha +1}\right) , \end{aligned}$$

which has been used for drawing the trajectories in Fig. 4.

The above mentioned region corresponds to $E_0\in [0,E^*(\alpha )]$ with

$$\begin{aligned} E^*(\alpha ) = E(\alpha -1,\alpha ) = (3\alpha +1)(\alpha -1) - 4\alpha (\alpha +1)\ln \left( \frac{2\alpha }{\alpha +1}\right) , \end{aligned}$$

where $E_0=0$ corresponds to the origin and $E_0=E^*(\alpha )$ to the stable manifold of the saddle. Among the trajectories connecting the segments $p+q=0$ and $q=0$, we need one which takes ‘time’ 1 / 2. To find an appropriate value of $E_0$, we compute $q<0$ from (29), substitute it in the first equation in (28), and integrate. This produces an equation for $E_0$:

30 $$\begin{aligned} I(E_0,\alpha ) = c\int _{p_0(E_0,\alpha )}^{p_1(E_0,\alpha )} \frac{\,\mathrm {d}p}{(1+\alpha /(1+p))\sqrt{E_0 - E(p,\alpha )}} = \frac{1}{2}, \end{aligned}$$

where $p(0)=p_0(E_0,\alpha )$ and $p(1/2)=p_1(E_0,\alpha )$ are the unique solutions of

$$\begin{aligned} E_0 = E(p_0,\alpha ) + p_0^2,\quad E_0 = E(p_1,\alpha ). \end{aligned}$$

Information about the range of $I(\cdot ,\alpha )$ is needed. For studying the limit as $E_0\rightarrow 0$, the Taylor expansion $E(p,\alpha ) = \frac{\alpha -1}{\alpha +1}p^2 + \mathcal {O}(p^3)$ and its consequence $p_0\approx \sqrt{E_0(\alpha +1)/(2\alpha )}$, $p_1\approx \sqrt{E_0(\alpha +1)/(\alpha -1)}$, imply

$$\begin{aligned} I(0,\alpha ) = \frac{c}{\sqrt{\alpha ^2 - 1}} \left( \frac{\pi }{2} - \arcsin \sqrt{\frac{\alpha - 1}{2\alpha }}\right) . \end{aligned}$$

As a consistency check, it is easily verified that the unique solution of the equation $I(0,\alpha )=1/2$ is the bifurcation value $\alpha _0$ defined by (16), (17). The uniqueness follows since $I(0,\alpha )$ is strictly decreasing as a function of $\alpha$, i.e.

$$\begin{aligned} I(0,\alpha ) < 1/2 \quad \text{ iff } \; \alpha > \alpha _0. \end{aligned}$$

On the other hand, by $p_1(E^*(\alpha ),\alpha ) = \alpha -1$ and $\partial _p E(\alpha -1,\alpha )$ the integrand in (30) loses its integrability as $E_0\rightarrow E^*(\alpha )$, implying

$$\begin{aligned} \lim _{E_0\rightarrow E^*(\alpha )} I(E_0,\alpha ) = \infty , \end{aligned}$$

which is actually obvious, since along the stable manifold of the saddle the critical point and (therefore the line $q=0$) cannot be reached in finite ’time’. These results and the continuity of $I(E_0,\alpha )$ complete the proof of the following proposition.

#### Proposition 6

Let $\alpha >\alpha _0$ as defined by (16), (17) and let $c(x)=\,$const. Then the equation (1) together with (*DBC*) has a non-trivial stationary solution.

#### Remark 3

Uniqueness of the steady state is equivalent to strict monotonicity of $I(E_0,\alpha )$ as a function of $E_0$. This will be proved indirectly by the decay result of the next section.

## Stability of nontrivial steady states

The main result of this section is that whenever a nontrivial steady state, as constructed (for certain cases) in the previous section, exists then it attracts all nontrivial solutions. We therefore make the assumption that there exists a stationary solution $({\bar{u}}(x),{\bar{v}}(x))$ of (1), satisfying

31 $$\begin{aligned}&\text{ either }\,\,\text{(PBC), } \quad ({\bar{u}},{\bar{v}}) \in {\mathcal {C}}^1(\mathbb {T}^1)^2,\quad \text{ and }\quad m \le {\bar{u}}(x),{\bar{v}}(x) \le M, \end{aligned}$$

32 $$\begin{aligned}&\quad \text{ or }\,\,\text{(DBC), } \quad ({\bar{u}},{\bar{v}}) \in {\mathcal {C}}^1([0,1])^2,\quad \text{ and }\quad m \le \frac{{\bar{u}}(x)}{x}, \frac{{\bar{v}}(x)}{1-x} \le M, \end{aligned}$$

for $x\in [0,1]$, with positive constants *m*, *M*.

For proving exponential convergence to nontrivial steady states, we need to strengthen the result of Proposition 2. Note that the following result is sharp in terms of the values of the parameter $\alpha$, proving uniform-in-time bounds away from zero for all parameter values above the bifurcation value, where the zero solution loses its stability.

### Lemma 2

Let the assumptions of Proposition 2 hold and let $\alpha >\alpha _0$, where $\alpha _0$ is the bifurcation value as defined in the preceding section. Then for (PBC) there exist $T,m>0$ such that

$$\begin{aligned} u(x,t), v(x,t)\ge m \quad \text {for }\; (x,t)\in [0,1]\times [T,\infty ). \end{aligned}$$

For (DBC) there exist $T,a>0$ such that for $(x,t)\in [0,1]\times [T,\infty )$

$$\begin{aligned}&u(x,t) \ge a\sin \left( b_0C\int _0^x \frac{\,\mathrm {d}y}{c(y)}\right) ,\quad v(x,t)\ge a\sin \left( b_0C\int _x^1 \frac{\,\mathrm {d}y}{c(y)}\right) . \end{aligned}$$

Furthermore, again for (DBC), there exists a constant $M>0$ such that

$$\begin{aligned} u(x,t) \le M x,\quad v(x,t) \le M(1-x),\quad \text {for } \; (x,t)\in [0,1]\times [T,\infty ). \end{aligned}$$

### Proof

(PBC): As a consequence of Proposition 2, there exist $m_0, T>0$, such that

$$\begin{aligned} u(x,T), v(x,T) \ge m_0 > 0 \quad \text{ for } \; x\in [0,1]. \end{aligned}$$

We choose

$$\begin{aligned} M = \frac{\underline{c}}{C}\min \left\{ m_0, \frac{\alpha -1}{2} \right\} >0, \end{aligned}$$

implying

$$\begin{aligned} U(X,T), V(X,T) \ge M \quad \text{ for } \; X\in [0,1]. \end{aligned}$$

Assume the bound holds for *V* for later times and consider the equation along characteristics for *U*:

$$\begin{aligned} \dot{U} = \frac{\alpha V}{1+\beta (U+V)} - U \ge \frac{\alpha M}{1 + \bar{\beta }(U+M)} - U. \end{aligned}$$

The right hand side is nonnegative for $U=M$ with the consequence $U\ge M$. Analogously for *V*. This completes the proof with $m = MC/{\bar{c}}$.

(DBC): By the result of Proposition 2, there exist $M>0$, $X_0\in (0,1)$, $t_2>t_1\ge 0$, such that $V \ge M$ in $[0,X_0]\times [t_1,t_2]$. Along *U*-characteristics staying inside this rectangle,

$$\begin{aligned} \dot{U}\ge \frac{\alpha M}{1+ \bar{\beta }(U+M)} - U \ge \frac{\alpha M}{2(1 + 2\bar{\beta }M)}, \end{aligned}$$

holds, as long as, furthermore,

$$\begin{aligned} U \le \min \left\{ M, \frac{\alpha M}{2(1 + 2\bar{\beta }M)}\right\} . \end{aligned}$$

For characteristics starting at $X=0$, $t=t^*\in [t_1,t_2)$, this implies

$$\begin{aligned} U \ge \frac{\alpha M}{2(1 + 2\bar{\beta }M)} (t-t^*) = \frac{\alpha M}{2(1 + 2\bar{\beta }M)} \,\frac{X}{C}, \end{aligned}$$

until

$$\begin{aligned} U = \min \left\{ M, \frac{\alpha M}{2(1 + 2\bar{\beta }M)}\min \left\{ 1,t_2-t^*, X_0/C \right\} \right\} . \end{aligned}$$

As a conclusion, there is a time $T\in (t_1,t_2]$ such that *U*(*X*, *T*) (and therefore also *u*(*x*, *T*)) increases (as a function of *X*) at least linearly away from $X=0$ up to a certain point, after which, by Proposition 2, it is bounded from below by a positive constant. This implies the existence of $A_0>0$, such that

$$\begin{aligned} U(X,T) \ge A_0 {\tilde{U}}(X),\quad V(X,T) \ge A_0 {\tilde{V}}(X), \end{aligned}$$

with ${\tilde{U}}, {\tilde{V}}$ defined by (16), (17), and the second inequality is proven analogously. Now we choose

$$\begin{aligned} A := \min \left\{ A_0, \frac{\alpha -\alpha _0}{2\alpha _0\bar{\beta }}\right\} , \end{aligned}$$

and assume $V(X,t)\ge A {\tilde{V}}(X)$ for $(X,t)\in [0,1]\times [T,\infty )$, in the equation for *U*. This implies

$$\begin{aligned} C\frac{\,\mathrm {d}U}{\,\mathrm {d}X} \ge \frac{\alpha A{\tilde{V}}}{1 + \bar{\beta }(U+A{\tilde{V}})} - U,\quad t\ge T. \end{aligned}$$

We claim that $U(X) = A{\tilde{U}}(X)$ is a subsolution for $t\ge T$, which follows from $A{\tilde{U}}(X) \le U(X,T)$ and

$$\begin{aligned} C\frac{\,\mathrm {d}(A{\tilde{U}})}{\,\mathrm {d}X}= & {} CAb_0 \cos (b_0 X) = -A \tan b_0\cos (b_0 X) \\= & {} \alpha _0 [A (\sin b_0\cos (b_0 X) - \sin (b_0 X)\cos b_0)] - A\sin (b_0 X) \\= & {} \frac{\alpha A {\tilde{V}}}{1 + (\alpha /\alpha _0-1)} - A{\tilde{U}} \le \frac{\alpha A{\tilde{V}}}{1 \!+\! 2\bar{\beta }A} - A{\tilde{U}} \le \frac{\alpha A{\tilde{V}}}{1 \!+\! \bar{\beta }(A{\tilde{U}} + A{\tilde{V}})} - A{\tilde{U}}. \end{aligned}$$

On the other hand, it can be proved analogously that $U \ge A{\tilde{U}}$ implies $V \ge A{\tilde{V}}$. This completes the proof of the lower bound with $a:= AC/\overline{c}$.

For the proof of the upper bound we observe that along characteristics

$$\begin{aligned} C\frac{\,\mathrm {d}U}{\,\mathrm {d}X} \le \frac{\alpha }{\underline{\beta }}, \end{aligned}$$

implying at most linear growth of *U* in terms of *X*, and therefore also of *u* in terms of *x*, with the analogous argument for *v*. $\square$

The convergence analysis is based on the Lyapunov functional

33 $$\begin{aligned} {\mathcal {H}}[u,v] := \frac{1}{2}\int _0^1 c\left( \frac{{\bar{v}}}{{\bar{u}}}(u-{\bar{u}})^2 +\frac{{\bar{u}}}{{\bar{v}}}(v-{\bar{v}})^2\right) \,\mathrm {d}x. \end{aligned}$$

Note that under the assumptions of Lemma 2 and with (31) or (32), the Lyapunov functional is well defined along the solution of (1) for $t\ge T$, since for (DBC) the integrand can be continuously extended by the value zero to $x=0,1$. For the computation of the time derivative of $\mathcal {H}[u,v]$ along solutions, the computation

$$\begin{aligned} -\int _0^1 c\frac{{\bar{v}}}{{\bar{u}}}&\left( u-{\bar{u}}\right) \partial _x(c u)\,\mathrm {d}x\\&=-\int _0^1 \frac{c{\bar{v}}}{c{\bar{u}}} \partial _x\frac{(c u-c {\bar{u}})^2}{2}\,\mathrm {d}x -\int _0^1 c\frac{{\bar{v}}}{{\bar{u}}}\left( u-{\bar{u}}\right) \partial _x(c {\bar{u}})\,\mathrm {d}x\\&= \int _0^1 \frac{(c u-c{\bar{u}})^2}{2} \,\frac{{\bar{u}} \partial _x(c{\bar{v}}) - {\bar{v}} \partial _x(c{\bar{u}})}{c {\bar{u}}^2}\,\mathrm {d}x -\int _0^1 c\frac{{\bar{v}}}{{\bar{u}}}\left( u-{\bar{u}}\right) \partial _x(c {\bar{u}})\,\mathrm {d}x\\&=\int c \frac{(u-{\bar{u}})^2}{2{\bar{u}}^2} \left( {\bar{u}} \partial _x(c{\bar{v}}) - {\bar{v}} \partial _x(c{\bar{u}})\right) \,\mathrm {d}x -\int _0^1 c\,\frac{{\bar{v}}}{ {\bar{u}}}\left( u-{\bar{u}}\right) \partial _x(c {\bar{u}})\,\mathrm {d}x \end{aligned}$$

will be used, where the derivatives on the right hand side can be eliminated by using the stationary equations. The integration by parts leading to the third line does not produce any boundary terms since ${\bar{v}}(u-{\bar{u}})^2/{\bar{u}}$ is periodic for (PBC) and the continuous extension vanishes at $x=0,1$ for (DBC). The analogous computation for the second part of $\mathcal {H}[u,v]$ leads to

$$\begin{aligned} \frac{\,\mathrm {d}}{\,\mathrm {d}t}\mathcal {H}[u,v] = -\frac{\alpha }{2}\int _0^1 \frac{c J(u,v,{\bar{u}},{\bar{v}})}{(1+u+v)(1+{\bar{u}}+{\bar{v}})}\,\mathrm {d}x, \end{aligned}$$

with

34 $$\begin{aligned} J(u,v,{\bar{u}},{\bar{v}}):= & {} \left( \frac{u-{\bar{u}}}{{\bar{u}}}\right) ^2 \left[ (1+u+v)\left( {\bar{u}}^2+{\bar{v}}^2\right) +2 {\bar{v}}^2 {\bar{u}}\right] \nonumber \\&+\left( \frac{v-{\bar{v}}}{{\bar{v}}}\right) ^2 \left[ (1+u+v)\left( {\bar{u}}^2+{\bar{v}}^2\right) +2 {\bar{u}}^2 {\bar{v}}\right] \nonumber \\&-2\frac{u-{\bar{u}}}{{\bar{u}}}\,\frac{v-{\bar{v}}}{{\bar{v}}} \left( {\bar{u}}^2+{\bar{v}}^2+{\bar{u}}^2{\bar{v}}+ {\bar{u}}{\bar{v}}^2\right) . \end{aligned}$$

Nonnegativity of *J* is not obvious, but actually even a coercivity property can be shown:

### Theorem 1

Let the assumptions of Proposition 2 hold, let $\alpha >\alpha _0$ with the bifurcation value $\alpha _0$, let a stationary solution $({\bar{u}},{\bar{v}})$ of (1) exist, which satisfies either (31) or (32), and let ${\mathcal {H}}[u,v]$ be defined by (33). Then there exists a constant $\gamma >0$ such that

$$\begin{aligned} \mathcal {H}[u,v](t) \le e^{-\gamma (t-T)}\mathcal {H}[u,v](T),\quad \text{ for } \; t\ge T, \end{aligned}$$

with *T* from Lemma 2.

### Proof

The representation (34) of *J* suggests the notation

$$\begin{aligned} {\hat{u}} := \frac{u-{\bar{u}}}{{\bar{u}}},\quad {\hat{v}} := \frac{v-{\bar{v}}}{{\bar{v}}}, \end{aligned}$$

with $({\hat{u}},{\hat{v}})\in [-1,\infty )^2$. This domain can be split into three subdomains, defined by ${\hat{u}}{\hat{v}} \le 0$, $0 < {\hat{u}}/{\hat{v}} \le 1$, and, respectively, $0< {\hat{v}}/{\hat{u}} <1$.

For ${\hat{u}}{\hat{v}} \le 0$, *J* is obviously nonnegative and satisfies

$$\begin{aligned} J\ge & {} \left( {\bar{u}}^2 + {\bar{v}}^2\right) \left( {\hat{u}}^2 + {\hat{v}}^2\right) = \frac{{\bar{u}}^2 + {\bar{v}}^2}{{\bar{u}} {\bar{v}}}\left( \frac{{\bar{v}}}{{\bar{u}}}(u-{\bar{u}})^2 + \frac{{\bar{u}}}{{\bar{v}}}(v-{\bar{v}})^2\right) \\\ge & {} 2\left( \frac{{\bar{v}}}{{\bar{u}}}(u-{\bar{u}})^2 + \frac{{\bar{u}}}{{\bar{v}}}(v-{\bar{v}})^2\right) . \end{aligned}$$

Now we consider the second case $0 < {\hat{u}}/{\hat{v}} \le 1$. The result for the third case then follows by symmetry. With $\lambda = {\hat{u}}/{\hat{v}}$, we introduce another change of variables $({\hat{u}},{\hat{v}}) \rightarrow (\lambda ,{\hat{v}})\in (0,1]\times [-1,\infty )$, giving

$$\begin{aligned} \frac{J}{{\hat{v}}^2}= & {} \lambda ^2 \left[ (1+{\bar{u}}+{\bar{v}} + ({\bar{u}}\lambda +{\bar{v}}){\hat{v}})\left( {\bar{u}}^2 + {\bar{v}}^2\right) + 2{\bar{u}} {\bar{v}}^2\right] \\&+ (1+{\bar{u}}+{\bar{v}} + ({\bar{u}}\lambda +{\bar{v}}){\hat{v}})\left( {\bar{u}}^2 + {\bar{v}}^2\right) + 2{\bar{u}}^2 {\bar{v}} \\&- 2\lambda \left( {\bar{u}}^2+{\bar{v}}^2+{\bar{u}}^2{\bar{v}}+ {\bar{u}}{\bar{v}}^2\right) \\= & {} \left( {\bar{u}}^2 + {\bar{v}}^2\right) ({\bar{u}}\lambda +{\bar{v}})\left( \lambda ^2 + 1 \right) ({\hat{v}} + 1) \\&+ (1-\lambda )\left[ (1-\lambda )\left( {\bar{u}}^2 + {\bar{v}}^2\right) + {\bar{u}}^3(\lambda ^2+1) + (1-\lambda )^2{\bar{u}} {\bar{v}}^2 + 2{\bar{u}}^2 {\bar{v}}\right] \\\ge & {} \left( {\bar{u}}^2 + {\bar{v}}^2\right) \left( {\bar{v}}({\hat{v}} + 1) + (1-\lambda )^2\right) . \end{aligned}$$

With one more set of variables, ${\tilde{u}} = {\hat{u}}\sqrt{{\bar{u}}{\bar{v}}} = \sqrt{{\bar{v}}/{\bar{u}}}(u-{\bar{u}})$, ${\tilde{v}} = {\hat{v}}\sqrt{{\bar{u}}{\bar{v}}} = \sqrt{{\bar{u}}/{\bar{v}}}(v-{\bar{v}})$, the above inequality is equivalent to

$$\begin{aligned} J\ge \frac{{\bar{u}}^2 + {\bar{v}}^2}{{\bar{u}} {\bar{v}}} \left( v {\tilde{v}}^2 + ({\tilde{u}} - {\tilde{v}})^2\right) \ge 2({\tilde{u}} - {\tilde{v}})^2 + \kappa {\tilde{v}}^2,\quad \text{ with } \kappa = \inf _{[0,1]} \frac{\left( {\bar{u}}^2 + {\bar{v}}^2\right) v}{{\bar{u}}{\bar{v}}}. \end{aligned}$$

The results of Lemma 2 and the assumption (31) or (32) imply that ${\bar{u}}^2 + {\bar{v}}^2$ and $v/{\bar{v}}$ are bounded from below and ${\bar{u}}$ is bounded from above, with the consequence $\kappa >0$, and therefore

$$\begin{aligned} J\ge \frac{2\kappa }{4+\kappa } \left( {\tilde{u}}^2 + {\tilde{v}}^2\right) = \frac{2\kappa }{4+\kappa }\left( \frac{{\bar{v}}}{{\bar{u}}}(u-{\bar{u}})^2 + \frac{{\bar{u}}}{{\bar{v}}}(v-{\bar{v}})^2\right) . \end{aligned}$$

With the common upper bound *M* for $u,v,{\bar{u}}$, and ${\bar{v}}$, the proof is completed with

$$\begin{aligned} \gamma = \frac{2\alpha \kappa }{(1+2M)^2(4+\kappa )}. \end{aligned}$$

$$\square $$

## Discussion

In this paper we derived, discussed and analyzed a mathematical model for the density of actin filament ends along the leading edge of a lamellipodium, a submodel of the filament based lamellipodium model (FBLM) (Manhart et al. 2015, 2016; Oelz and Schmeiser 2010). The main modeling assumption is that the actin network can be described by two families of filaments that point to the left and to the right respectively. This is supported by the observation that the angle distribution of filaments with respect to the leading edge has two prominent peaks at $\pm 35^\circ$ (Winkler et al. 2012; Maly and Borisy 2001). The equations for the two families consist of a transport term describing the movement of filaments to the left and right by lateral flow, which is a consequence of actin polymerization and the angle of the filaments with respect to the leading edge, i.e., the geometry of the lamellipodium. In this work the lateral flow is assumed to be given, however in the full FBLM it is implicitly determined by the full dynamics of the system. The creation and degradation of the filaments are described by branching and capping processes, consistent with the biological knowledge available (Machesky and Insall 1998; Mullins et al. 1998; Svitkina and Borisy 1999; Weeds and Maciver 1993).

A particular emphasis is put on the branching rate, which is assumed to be limited by the local availability of activated Arp2 / 3 at the membrane. The hypothesis that active Arp2 / 3 is localized at the membrane is supported the fact that it is activated by proteins of the WASP/Scar family, which in turn can interact with transmembrane receptors (see Machesky and Insall 1998; Pollard et al. 2000). This hypothesis has already been used in several other models such as in Grimm et al. (2003) and Atilgan et al. (2005), the latter also specifies experimental setups to test Arp2/3 localization.

**Fig. 5:**
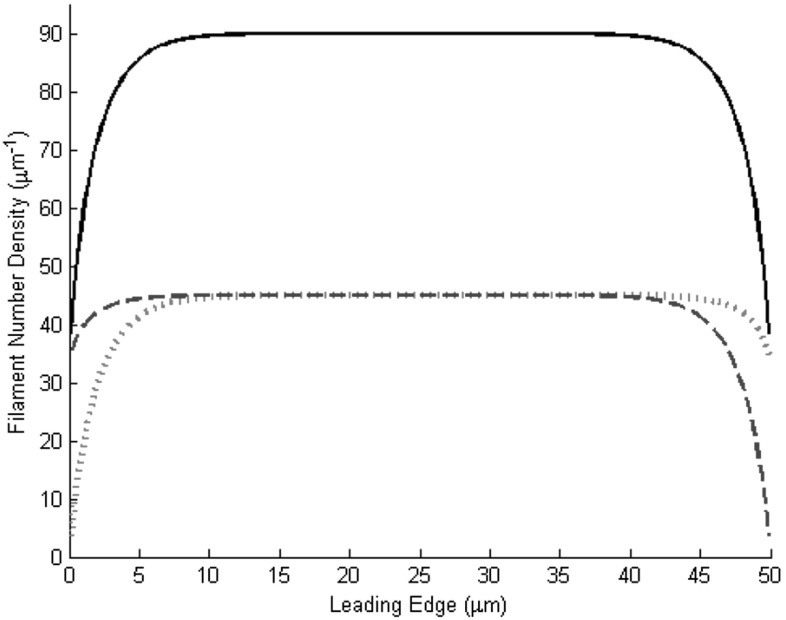
Density distribution of filaments along the leading edge for Dirichlet boundary conditions as given in Eq. (2) using the equation prior to non-dimensionalization. *Red-dotted* and *blue-dashed lines* refer to *right* and *left* moving filaments respectively; the *black-solid line* shows the total filament end density. Parameters (not scaled): $L=50\, \upmu \mathrm{m}$, $\kappa _\text {br}=10 / \mathrm{s}$, $\kappa _\text {cap}=0.1\,/\mathrm{s}$, $c_\text {rec}=9.1\,/(\upmu \mathrm{m}\,\mathrm{s})$, $c(x)\equiv 0.305\, \upmu \mathrm{m/s}$

We assume here that once activated, Arp2 / 3 is quickly incorporated into the branches of nearby actin filaments. In Grimm et al. (2003) this *local* scenario of how the availability of Arp2 / 3 effects the branching rate, has been compared to a *global* one, in which activated Arp2 / 3 spreads evenly along the leading edge before being incorporated into branches. The local model gave much better agreement with biological measurements of the barbed end distribution (i.e., a rather flat distribution with steep drops at the sides, see below). It should also be noted that the model of this paper and the local model of Grimm et al. (2003) differ in the nature of the nonlinearity in the branching term: in Grimm et al. (2003) it is proportional to $v/(u+v)$ and $u/(u+v)$ for the left and right moving family respectively, whilst ours is proportional to $v/(1+u+v)$ and $u/(1+u+v)$ respectively. This implies that if locally e.g. *u* is zero, its branching rate is constant and independent of the number of filaments of the other family, whilst in our model it will be proportional to $v/(1+v)$. The different shape also has a big impact on the mathematical analysis of the models.

The mathematical description of actin polymerization and in particular modeling of branching and capping events has received a lot of attention. Whilst the model presented in this paper acts only along the membrane, there exist also several models in 2D (e.g., in Maly and Borisy (2001) an emphasis is put on the angle between the filaments and the membrane) and 3D (e.g., in Atilgan et al. (2005) and Schmeiser and Winkler (2015) the interplay between geometry and actin polymerization has been examined).

The description of the filament end density is complemented by two types of biologically interesting boundary conditions: periodic boundary conditions describe cells, which are surrounded by a lamellipodium. This situation typically appears for stationary, or almost stationary cells. Dirichlet boundary conditions, i.e. zero influx of right moving filaments at the left, and left moving filaments at the right, are appropriate when referring to cells, where the lamellipodium is located only on one side. Examples for both cases are several types of fibroblasts (see Small et al. 2002; Yam et al. 2007; Mogilner and Keren 2009 an references therein). The best studied cell type for both situations, however, are keratocytes due to their persistent movement and regular shape (Small et al. 1995; Vallotton et al. 2005).

The mathematical analysis presented shows, that if the branching rate is small compared to the capping rate, in particular if $\alpha =\kappa _\text {br}/ \kappa _\text {cap}<1$, the densities of both filaments will converge to zero. On the other hand if $\alpha$ is large enough, the model will converge exponentially to a stable, non-zero density distribution. This is biologically very relevant, since for the cell types under discussion, especially keratocytes, the actin distributions observed were often very stable over time. This suggests that the actin distribution obtained are determined by the cell’s internal biochemical state and will return to its original shape after perturbations. Comparing this to moving keratocytes, where most data is available, the parameter values available in literature, suggest $\kappa _\text {br}$ to be of the order of tens of $\mu m/s$ and $\kappa _\text {cap}$ to be of the order of tenths to a few $\mu m/s$ (see Grimm et al. 2003; Pollard et al. 2000), i.e., these cells are always in the regime leading to stable, non-zero filament densities. As to the shape of the distribution itself, Fig. 5 shows the outcome of a simulation of the model using Dirichlet boundary conditions and a constant lateral flow rate (see the caption of Fig. 5 for parameter values). The (non-scaled) lateral flow speed has been chosen to be consistent with the lateral flow of a filament having an angle of $35^\circ$ with the leading edge in a cell moving at $15\,\upmu \mathrm{m}$/min, a typical speed for keratocytes (Small et al. 1995). The distribution depicted shows qualitative agreement with density distributions measured along the leading edge which were reported in Grimm et al. (2003), i.e., a rather flat distribution with a steep drop at the sides. It should be noted that also other, less flat density distributions have been observed (Keren et al. 2008), which are also reproducible with our model. Future experimental results, both in terms of determining rate constants and measuring barbed end densities, are necessary to help further validate the model.

The positivity result presented in Proposition 2 suggests a mechanism of how a cell can recreate a full lamellipodium from a small number of filaments, a situation which has been observed experimentally, for example in the context of intracellular wound healing (Vinzenz et al. 2012).

Mathematically the stability result of Theorem 1 answers the question of long-term behavior, however under the premise that a non-trivial steady state exists. This was only proven for constant lateral flow [for (PBC) and (DBC)] and for almost constant lateral flow speed [for (PBC)]. For both types of boundary condition the existence of a non-trivial steady state larger than and near the bifurcation value $\alpha _0$ is a consequence of the bifurcation results of Proposition 4. Even though the existence of non-trivial steady states for all $\alpha >\alpha _0$ and non-constant lateral flow is likely, it remains to be proven. Finally, also the expected (exponential) convergence to zero for Dirichlet boundary conditions for $\alpha \in [1, \alpha _0]$ is left for future work.
